# CHEK2 Mutation in Patient with Multiple Endocrine Glands Tumors. Case Report

**DOI:** 10.3390/ijerph17124397

**Published:** 2020-06-18

**Authors:** Anna Szeliga, Aleksandra Pralat, Wiktoria Witczak, Agnieszka Podfigurna, Cezary Wojtyla, Anna Kostrzak, Blazej Meczekalski

**Affiliations:** 1 Department of Gynecological Endocrinology, Poznan University of Medical Sciences, 61-701 Poznan, Poland; annamaria.szeliga@gmail.com (A.S.); agnieszkapodfigurna@gmail.com (A.P.); ankos30@op.pl (A.K.); 2 Students’ Scientific Society of the Department of Gynecological Endocrinology, Poznan University of Medical Sciences, 61-701 Poznan, Poland; panikukulska@gmail.com (A.P.); wwitczak1@wp.pl (W.W.); 3 International Prevention Research Institute—Collaborating Centre, State University of Applied Sciences, 62-800 Kalisz, Poland; czwo@op.pl; 4 Department of Oncological Gynecology and Obstetrics, Center of Postgraduate Medical Education, 00-416 Warsaw, Poland

**Keywords:** CHEK2, MEN, Cushing syndrome, hypertension, multiple endocrine glands tumors

## Abstract

Background: Many studies show the occurrence of several multiple endocrine neoplasia syndromes caused by different mutations, for example, in MEN1 and RET genes. Nevertheless, there are less common mutations causing multiple endocrine glands tumors. Examples of such mutations are CHEK2 gene mutations, causing breast, kidney, gastric, colorectal, prostate, lung, ovarian, and thyroid cancers. Case description: In 2005, a 30-year-old woman was admitted to the hospital due to uncontrolled hypertension and obesity. Performed tests have shown ACTH (adrenocorticotropic hormone)—independent micronodular adrenal hyperplasia (AIMAH) as a cause. In 2010, the further diagnostic analysis revealed Cushing’s disease caused by ACTH-secreting pituitary microadenoma. Additionally, in 2011, the patient underwent the strumectomy of multinodular struma. Papillary thyroid carcinoma was found in the excised tissue. In 2018, transvaginal ultrasonography revealed a tumor of the right ovary. After a performed hysterectomy with bilateral salpingo-oophorectomy, the histopathology result has shown female adnexal tumors of probable Wolffian origin (FATWO) located in the broad ligament of the uterus. Due to the history of multiglandular diseases, the patient was referred to genetic testing. We found a positive pathogenic mutation in CHEK2-suppressor gene involved in DNA repair, cell cycle arrest, and apoptosis in response to DNA damage. Conclusion: CHEK2 variants may predispose to a range of endocrine glands tumors, including those identified in our patient. Multiple endocrine glands tumors, as in the presented patient, are a serious problem of public health, due to numerous hospitalizations and necessary repeated surgical treatments. Moreover, the association between CHEK2 and ovarian cancer can be a serious problem with reproductive health.

## 1. Introduction

The term multiple endocrine neoplasia (MEN) encompasses several distinct syndromes featuring tumors of endocrine glands, each with its characteristic pattern. MEN type 1 describes the association of pituitary, parathyroid, and pancreatic islet cells [1]. In contrast, MEN type 2 (MEN 2) condition is associated with pheochromocytoma, medullary thyroid carcinoma, hyperparathyroidism, mucosal and digestive neurofibromatosis, and Marfanoid habitus [1]. Mutations in the PRKAR1A and KIT genes predispose to rare Carney’s syndrome. Except for the triad of diseases—gastric stromal sarcoma, pulmonary chondroma, and paraganglioma, they can also cause micronodular adrenal hyperplasia, pituitary adenomas, and ovarian and thyroid tumors [2]. Tumor-associated mutations in the PTEN gene may cause Cowden’s syndrome, in which multinodular struma, follicular thyroid cancer, uterine, and genitourinary tract tumors are observed [3]. CHEK2 gene encodes checkpoint kinase 2, which is a serine/threonine kinase activated upon DNA damage and is implicated in pathways that regulate DNA repair, cell cycle arrest (in gap 1 phase), or apoptosis in response to the initial damage. When activated, the encoded protein is known to inhibit CDC25C phosphatase, preventing entry into mitosis, and has been shown to stabilize the tumor suppressor protein p53 [4]. Loss of kinase function caused by mutations in the CHEK2 gene has been linked with Li–Fraumeni syndrome, sarcomas, breast, and colorectal cancer, brain, thyroid, lung, ovary, bladder, and prostate and kidney tumors [4]. In Poland, there are three polymorphic variants of CHEK2, which are present in 5.5% of the population [5]. The missense CHEK2 I157T allele was found in 4,8% of controls, and it was more common in cancer cases than in controls—the association was significant at the *p* ≤ 0.01 level [5]. Studies reported no significant difference in the survival of the p.I157T carriers and non-carriers [6]. To summarize, CHEK2 is a multisite cancer gene that increases the risk of several cancers.

In this case report, we present a 44-year-old woman with multiple malignancies of endocrine glands without a pattern characteristic for described syndromes, caused by CHEK2 mutation. Multiple endocrine glands tumors, as in the presented patient, are a serious problem of public health due to numerous hospitalizations, necessary repeated surgical treatments, and quality of life [7].

## 2. Case Report

In 2005, a 30-year-old woman was admitted to the Department of Endocrinology, Poznan University of Medical Sciences, Poznan, Poland, with uncontrolled hypertension and obesity. On physical examination at admission, the patient was 163-cm tall and weighed 87 kg, with a body mass index (BMI) of 33 kg/m^2^. The mean arterial pressure in daily measurements was 140/105 mmHg. In the clinical examination, Cushing syndrome was suspected. In the hormonal evaluation, elevated cortisol concentration with the normal circadian rhythm of cortisol secretion was found.

Nevertheless, in a dexamethasone suppression test, the patient presented an elevated cortisol concentration after administration of 1 mg of dexamethasone (Table 1). Serum and diurnal urine concentration of Na, K, Ca, and phosphates were normal. The serum glucose profile is presented in Table 2.

Among the complications of hypertension, the patient revealed a hypertensive retinopathy of stage I/II.

We also performed dual-energy X-ray absorptiometry: T-score at L1–L4 lumbar spine was −1.57, and the patient was diagnosed with osteopenia.

Due to adrenocorticotropic hormone (ACTH)-independent hypercortisolemia, we performed diagnostic imaging; an adrenal computed tomography (CT) scan revealed the presence of moderately enlarged left gland presenting homogeneous absorption with no focal changes, whereas the right adrenal gland appeared normal. Magnetic resonance imaging (MRI) did not confirm any lesions in the pituitary gland. The biological and radiological data were highly suggestive of ACTH-independent Cushing’s syndrome. Surgical removal of the left adrenal gland was performed. Histopathology results showed micronodular adrenal hyperplasia. The patient was prescribed indapamide 1.5 mg once daily and perindopril 5 mg once daily. Moreover, sodium and potassium, calcium, and vitamin D3 supplementation were prescribed.

Despite surgical treatment, in 2010, the patient was admitted to the hospital due to recurrent uncontrolled hypertension. Her BMI was 35.8 kg/m², and mean arterial pressure in daily measurements was 155/120 mmHg. Skin hyperpigmentation was observed. The hormonal profile is presented in Table 3 and the patient’s blood glucose level before treatment in 2010 is presented in Table 4.

Adrenal angiography did not show any focal changes. MRI of the sella region was therefore performed, revealing a hypointense pituitary tumor of 5 mm in diameter located in the left anterior pituitary lobe. These MRI findings were highly suggestive of pituitary microadenoma. 

Trans-sphenoidal total resection of the pituitary tumor was performed. Upon immunohistochemical examination, the resected pituitary microadenoma cells exhibited positive staining for the ACTH antibody. The patient was prescribed perindopril 5 mg once daily, losartan 50 mg once daily, spironolactone 50 mg once daily, bisoprolol 5 mg once daily, indapamide 1.5 mg once daily, acetylsalicylic acid 75 mg once daily, desmopressin 0.1 mg twice daily, and hydrocortisone 20 mg–10 mg–0 mg daily.

Subsequently, in 2011, the patient was admitted to the Department of Surgery for a scheduled strumectomy due to multinodular struma, which had been diagnosed in 2010. During the physical examination, an enlarged and fibroelastic thyroid was identified. The serum concentration of thyroid-stimulating hormone (TSH), free triiodothyronine (fT3), and free thyroxine (fT4) were normal, indicating euthyreosis. The hormonal profile is presented in Table 5.

Ultrasound imaging demonstrated multiple nodules in both thyroid lobes, with the biggest solid nodule with calcification and hypoechoic outline measuring 1.17 × 1.82 cm.

The patient underwent a total strumectomy. Histological sections from the left lobe showed features of papillary thyroid carcinoma. The lesion was 1 cm in diameter with hyaline-fibrous capsule and calcification inside. Histopathology also revealed a multinodular struma of the right lobe. The patient was prescribed levothyroxine 100 µg (1 × 1), and she was qualified for iodine treatment.

In 2018, the patient was admitted to the Department of Gynecological Endocrinology Poznan University of Medical Sciences, Poznan, Poland, due to irregular menstrual cycle.

In the transvaginal ultrasonography, multiple small uterine fibroids were revealed. The endometrial thickness was 7 mm. The right ovary was enlarged by a cystic-solid, unilocular tumor with a size of 97 mm × 69 mm with central blood flow. There were no abnormalities in the left ovary revealed in the ultrasound examination.

Additionally, a hormonal analysis was performed (Table 6).

Due to ultrasound features and elevated Risk of Ovarian Malignancy Algorithm (ROMA) results, the patient was qualified for surgical treatment. Hysterectomy with bilateral salpingo-oophorectomy and retroperitoneal tumor excision were performed. Histopathology results have shown uterine glandular polyp (0.5 cm), serous superficial papillomas and superficial endometriosis in both ovaries, and female adnexal tumor of probable Wolffian origin (FATWO) located in the broad ligament of the uterus. The FATWO was 8 cm in diameter, contained a variable admixture of diffuse, solid, and sieve-like cystic areas filled with blood. Macroscopically, the tumor was soft and uncapsulated. Immunohistochemical examination revealed an interesting profile of this tumor: ER(+), PgR(+), AR(+), GATA3(+), calretinin(+), HBME(+), WT1(+), cytokeratins AE1/AE3(+), CK7(+), CKAE 1/3 (+), melan A(+), α-inhibin(+), CD99 (+), CEA mono(−), PAX8(−), TTF1(−), RCC(−), CK20(−), CD117(−), S100(−), EMA(−), HMB45(−), and Ki67 (5%).

Due to a history of multiglandular diseases: micronodular adrenal hyperplasia, pituitary adenoma, papillary thyroid carcinoma, and ovarian tumor, the patient was referred to genetic testing for mutations associated with multiple gland malignancy. We performed an analysis of 83 genes deletion/duplication (Invitae Multi Cancer Panel) in the whole blood sample. The Invitae Multi-Cancer Panel analyzes 83 genes associated with hereditary cancers across major organ systems, including: breast and gynecologic (breast, ovarian, uterine); gastrointestinal (colorectal, gastric, pancreatic); endocrine (thyroid, paraganglioma/pheochromocytoma, parathyroid, pituitary); genitourinary (renal/urinary tract, prostate); skin (melanoma, basal cell carcinoma); brain/nervous system sarcoma and hematologic (myelodysplastic syndrome/leukemia) cancers: AIP; ALK; APC; ATM; AXIN2; BAP1; BARD1; BLM; BMPR1A; BRCA1; BRCA2; BRIP1; CASR; CDC73; CDH1; CDK4; CDKN1B; CDKN1C; CDKN2A; CEBPA; CHEK2; CTNNA1; DICER1; DIS3L2; EGFR; EPCAM; FH; FLCN; GATA2; GPC3; GREM1; HOXB13; HRAS; KIT; MAX; MEN1; MET; MITF; MLH1; MSH2; MSH3; MSH6; MUTYH; NBN; NF1; NF2; NTHL1; PALB2; PDGFRA; PHOX2B; PMS2; POLD1; POLE; POT1; PRKAR1A; PTCH1; PTEN; RAD50; RAD51C; RAD51D; RB1; RECQL4; RET; RUNX1; SDHA; SDHAF2; SDHB; SDHC; SDHD; SMAD4; SMARCA4; SMARCB1; SMARCE1; STK11; SUFU; TERC; TERT; TMEM127; TP53; TSC1; TSC2; VHL; WRN; and WT1 gene mutations. We received positive results—pathogenic variant, exon 4. c.470T > C (p.Ile157Thr) heterozygous, with low penetrance identified in CHEK2, and a variant, exon 17. c.2484C > T (silent) heterozygous, of uncertain significance identified in KIT. Both mutations are not associated with MEN1 or MEN2 syndromes.

## 3. Discussion

The c.470T > C (p.Ile157Thr) variant in the CHEK2 gene is classified as a pathogenic variant with low penetrance. The isoleucine residue is moderately conserved, and there is a moderate physicochemical difference between isoleucine and threonine. Experimental studies find that this missense change reduces the binding of CHEK2 protein to Cdc25A, BRCA1, and p53 proteins in vitro and may have a dominant-negative effect on cells. However, it does not affect CHEK2 protein kinase activity [8]. The relationship between these experimental findings and cancer risk is unclear. Patients presenting CHEK2 mutations present a predisposition to certain cancers. The lifetime risk of breast cancer in females with a single pathogenic CHEK2 variant is 25–39% [9]. The risk was found to be more pronounced for lobular type breast tumors [10]. This variant has low penetrance because it does not confer the same level of cancer risk as other CHEK2 pathogenic variants. There is also a reported association between CHEK2 and other cancers, including male breast, kidney, gastric, colorectal, prostate, lung, ovarian, and thyroid cancers. Still, the lifetime risk of these other cancers is unknown [5,11,12,13]. Biological relatives have a chance of being at risk for CHEK2-related conditions and should consider testing if clinically appropriate.

A variant of uncertain significance, c.2484C > T (silent), was identified in KIT. This sequence change affects codon 828 of the KIT mRNA. It is a “silent” change, meaning that it does not change the encoded amino acid sequence of the KIT protein. This variant has not been reported in individuals with KIT-related diseases. The KIT gene is associated with autosomal dominant piebaldism [14], gastrointestinal stromal tumors (GISTs) [15], and familial mastocytosis [16]. The clinical significance of this result is uncertain. In summary, the available evidence is currently insufficient to determine the role of this variant in disease. Therefore, it has been classified as a Variant of Uncertain Signification.

Genetic testing was performed for our patient, and it was positive for the pathogenic variant c.470T > C (p.Ile157Thr) with low penetrance identified in CHEK2 and a variant c.2484C > T (silent) of uncertain significance identified in KIT. KIT mutation, as a silent one, is not suspected of being a cause of the patient’s clinical condition. On the other hand, in the described patient, we identified CHEK2-associated typical tumors, like papillary thyroid cancer.

It was reported that the CHEK2 mutation was seen in 73 of 468 (15.6%) unselected patients with papillary thyroid cancer, compared to 28 of 460 (6.0%) age—and sex-matched controls (OR 3.3; *p* < 0.0001) [17]. On the other hand, it was a truncating mutation (IVS2 + 1G > A, 1100delC or del5395) that was associated with a higher risk of thyroid cancer (OR  =  5.7; *p*  =  0.006), than was observed in the missense mutation I157T (OR  =  2.8; *p*  =  0.0001) [15], which we identified in the patient. The patient was also diagnosed with micronodular adrenal hyperplasia. This disease’s correlation with a mutation in CHEK2 is uncertain. It is known that CHEK2 is a TP53-associated gene [8]. Because of that, they are similarly linked to Li–Fraumeni syndrome and presumably hyperplasia and tumors of adrenal glands [18]. Moreover, the patient was suffering from pituitary microadenoma. CHEK2 mutations have also been found in some brain tumors, such as meningioma or glioma [19].

The patient was also diagnosed with a uterine glandular polyp and FATWO. Endometrial polyps carry a small risk of cancer. The risk of endometrial cancer in women with endometrial polyps is 1.3% [20]. While mutation in CHEK2 has been reported in patients with endometrial cancers [5], and because of that, it is worth considering if CHEK2 mutation affected our patient’s uterine polyps. In the literature, we found information indicating that positive associations were seen with the CHEK2 I157T missense variant and ovarian cystadenomas (OR = 1.7; *p* = 0.005), borderline ovarian cancers (OR = 2.6; *p* = 0.002), and low-grade invasive cancers (OR = 2.1; *p* = 0.04) [21]. All these data indicate that CHEK2 variants may predispose to a range of ovarian tumor types of low malignant potential. It is difficult to refer to the association between FATWO and CHEK2-related cancers, because of its rarity.

FATWO is a very rare gynecologic neoplasm of low malignant potential, with fewer than 90 cases being reported worldwide [22]. It is presumed to be derived from the mesonephric (Wolffian) duct remnants in the upper female genital tract [23]. Moreover, some recurrent and metastatic cases have been reported in approximately 11% of cases [24]. Tumor-defining molecular alterations have yet to be discovered. FATWO originates from mesonephric (Wolffian) duct remnants. Tumors are present along the trajectory of the Wolffian duct: from the ovarian hilum, along the mesosalpinx, broad ligament, lateral aspects of the uterus and cervix, to the outer third of the vagina. There are no disease-specific mutations or genetic alterations identified [25]. Many patients remain asymptomatic, and the tumors are discovered incidentally [22]. Sometimes patients can present lower abdominal pain or distention, pelvic mass, abnormal vaginal bleeding, or ascites [23]. In most cases, tumors are not hormonally active. In rare cases, hormonally active tumors can cause endometrial hyperplasia [26]. Most tumors are unilateral, and rarely bilateral. On diagnostic imaging, FATWO are usually well defined, encapsulated, lobulated masses. As in our case, they may demonstrate solid and cystic areas. In an MRI, they present with low signal intensity rim around the tumor on T2 weighted MRI images [27,28]. Although most cases of FATWO are benign, a few aggressive cases occur [24].

Due to the found mutation, screening, and prevention of breast and colorectal cancers was implemented in the case of our patient, due to multiple neoplasia in the past. The patient is only 44 years old, and the lifetime risk of breast cancer in females with a single pathogenic CHEK2 variant is 20–44% [13]. Interestingly, mutations of CHEK2 have been associated with resistance to anthracycline-based chemotherapy in patients with breast cancer [29]. Significant associations of the CHEK2 I157T variant with colorectal cancer susceptibility were found (OR = 1.67, 95% CI = 1.24–2.26, *p* = 0.0008) [13]. There are limited guidelines for screening and prevention of CHEK2-related breast and colorectal cancers.

According to the correlation between CHEK2 and TP3 genes and the role of CHEK2 (suppressor gene that is involved in DNA repair, cell cycle arrest, or apoptosis in response to DNA damage)—mutations to the CHEK2 gene have been labeled as causes to a wide range of cancers. Thus, micronodular adrenal hyperplasia, pituitary microadenoma, papillary thyroid cancer, uterine, ovarian, and retroperitoneal (FATWO) lesions are possible after-effects of CHEK2 missense mutation identified in the patient.

## 4. Conclusions

The described case is undoubtedly significant and important for gynecologists, endocrinologists, geneticists, and other practitioners. It is not clearly established whether there is a significant link between a constellation of tumors presented in a described patient and the mutation found in the CHEK2 gene. Further evaluation of the neoplastic risk associated with CHEK2 gene mutation is necessary. 

In conclusion, we want to underline that in cases of multiple endocrine neoplasias, thought should be directed at genetic testing and mutations. Additionally, possible predispositions and syndromes should be considered, because they may change how the patient is monitored clinically. Multiple endocrine glands tumors, as in the presented patient, are a serious problem of public health due to multiple hospitalizations and necessary repeated surgical treatments. Moreover, the association between CHEK2 and ovarian cancer can be a serious problem with reproductive health.

## Figures and Tables

**Table 1:** Concentrations of chosen hormones in the described patient in 2005.

Hormonal profile in 2005	**Hormone**	**Result**	**Normal Range**
	ACTH	27.7 pg/ml	0–50 pg/ml
	ACTH after 1 mg of dexamethasone	4.4 pg/ml	
	Cortisol at 8 am	1354–1151–949.8 nmol/l	220–690 nmol/l
	Cortisol at 6 pm	967.9 nmol/l	50–165 nmol/l
	Cortisol at 8 pm	503 nmol/l	
	Cortisol after 1 mg dexamethasone	120.5 nmol/l	

ACTH, adrenocorticotropic hormone.

**Table 2:** Patient’s blood glucose level before treatment in 2005.

Time of Measurement	8 AM	11 AM	5 PM	8 PM
Blood glucose concentration [mg/dl]	78	111	94	118

**Table 3:** Concentrations of chosen hormones in the described patient in 2010.

Hormonal profile	**Hormone**	**Result**	**Normal Range**
	ACTH at 8 am	47.6 pg/ml	<60 pg/ml
	ACTH at 6 pm	47.35 pg/ml	
	Cortisol at 8 am	26.7 µg/dl	4.2–38.4 µg/dl
	Cortisol at 6 pm	25.8 µg/dl	1.7–16.6 µg/dl
	Cortisol in 24-h urine collection	359.304 µg/d	32–243 µg/d

ACTH, adrenocorticotropic hormone.

**Table 4:** Patient’s blood glucose level before treatment in 2010.

Time of Measurement	8 AM	11 AM	5 PM	8 PM
Blood glucose concentration [mg/dl]	99.4	86.4	102.6	91.8

**Table 5:** Concentrations of chosen hormones in the described patient.

Hormone	Result	Normal Range
TSH	1.97 µIU/ml	0.5–4.8 µIU/ml
fT3	3.26 pg/ml	2.3–4.2 pg/ml
fT4	1.22 ng/dl	0.8–2.3 ng/dl

TSH, thyroid-stimulating hormone; fT3, free triiodothyronine; fT4, free thyroxine.

**Table 6:** Hormonal analysis.

Hormone or Biochemical Marker	Result	Normal Range
FSH	6.05 mIU/ml	3.5–12.5 mIU/ml
LH	4.91 mIU/ml	1.4–9.6 mIU/ml
Estradiol	40.95 pg/ml	12.4–233 pg/ml
Prolactin	5.6 ng/ml	5–25 ng/ml
Testosterone	0.08 ng/ml	0.06–0.82 ng/ml
DHEA-S	1.53 µmol/l	0.96–6.95 µmol/l
HE4	72.21 pmol/l	<150 pmol/l
CA125	71.4 U/ml	<35 U/ml
ROMA	17.55% (high risk)	<11.4%

FSH, follicle-stimulating hormone; LH, luteinizing hormone; DHEA-S, dehydroepiandrosterone sulfate; HE4, human epididymis protein 4; CA125, cancer antigen 125; ROMA, Risk of Ovarian Malignancy Algorithm.
